# Different Experiences of Adult Child and Spousal Caregivers with Family Conflict

**DOI:** 10.1093/geroni/igab046.2921

**Published:** 2021-12-17

**Authors:** Stacy Yun, Kendall Weber, Geffen Ferszt, JoAnna Dieker, Sara Qualls

**Affiliations:** University of Colorado Colorado Springs, Colorado Springs, Colorado, United States

## Abstract

Previous research indicates that different types of caregivers report distinct levels of family conflict (Dieker et al., 2019). However, as half (52%) of the participants in the previous study did not report family conflict, the purpose of this study was to examine the relationships among types of caregivers, family conflict, and caregiver burden in those who experience family conflict. Participants (N = 277; aged 19 to 87; M = 52.96) comprised of 197 adult child and 80 spousal caregivers. They completed the Caregiver Reaction Scale (O’Malley & Qualls, 2017) to assess different aspects of the caregiving experience. Hierarchical regressions (block 1: demographics, block 2: family conflict) were computed to predict caregiving burden. For spousal caregivers, the final model explained 2.2% of the variance in caregiving burden, F(7, 69) = 0.22, p = .98. None of the variables were significant. Additionally, family conflict did not uniquely influence caregiving burden beyond demographics, Fchange(2, 69) = 0.03, p = .97. For adult child caregivers, the set of predictors accounted for 20.5% of the variance in caregiving burden, F(7, 183) = 6.75, p < .001. Having more family beliefs and support conflict predicted greater caregiving burden (ps < .01). Family conflict scores also significantly explicated caregiver burden beyond demographics, Fchange(2, 183) = 17.60, p < .001. Results suggest that family conflict is a stronger driver of caregiver burden for adult child than spousal caregivers. Findings imply the need for clinicians to target appropriate interventions for adult child caregivers to reduce caregiver burden.

